# Closed-Incision Negative-Pressure Wound Therapy in Proximal and Distal Femur Megaprosthetic Reconstructions after Bone Tumor Resections

**DOI:** 10.1055/s-0045-1802965

**Published:** 2025-04-28

**Authors:** Joaquim Soares do Brito, Rodrigo Cardoso, Rodrigo Goes, André Spranger, Paulo Almeida, José Portela

**Affiliations:** 1 Departamento de Ortopedia e Trauma, Hospital de Santa Maria, Unidade Local de Saúde Santa Maria,Lisboa, Portugal; 2 Grupo de Oncologia Ortopédica, Instituto Nacional de Traumatologia e Ortopedia, Rio de Janeiro, Brasil; 3 Grupo de Oncologia Ortopédica, Hospital Universitário Gaffre Guinle, Universidade Federal do Estado do Rio de Janeiro (UNIRIO), Rio de Janeiro, Brasil

**Keywords:** negative-pressure wound therapy, neoplasms, prosthesis, sarcoma, surgical wound infection

## Abstract

**Objective** Surgical management for bone tumors is aggressive in nature and frequently followed by wound-related complications (WRCs). To minimize these events, different strategies have been employed, with closed-incision negative-pressure wound therapy (ciNPWT) emerging as a potential adjuvant. With this study we intend to assess the impact of this technique in minimizing WRCs in patients with proximal and distal femur tumors treated with megaprosthesis.

**Methods** This was an observational retrospective study including 41 participants diagnosed with proximal or distal femur tumors treated with wide resection and reconstruction using a megaprosthesis. Patients were divided into two groups based on the postoperative surgical dressing applied: the vacuum-assisted closure group (VAC) received ciNPWT; and the non-VAC group that received conventional dressings. Data regarding postoperative WRCs and other potential variables of interest were recorded. Statistical analysis was carried out using the IBM SPSS Statistics, version 24.0.

**Results** There were 20 patients included in the VAC and 21 in the non-VAC group. The majority of patients presented no complications and there were no differences between groups in terms of WRCs, including infection. Nonetheless, wound dehiscence and persistent fluid leakage had a positive correlation with the diagnosis of infection, which all together presented correlation with the need for surgical revision.

**Conclusion** Despite the absence of statistical significance, ciNPWT seems to help minimize wound dehiscence, persistent wound leakage and surgical site infections in patients with proximal and distal femur bone tumors treated with megaprosthesis. Also, wound dehiscence and persistent wound leakage correlate well with surgical site infection, and those three parameters correlate with the need for surgical revision.

## Introduction

Bone reconstruction with megaprosthesis is a common approach after tumor resection or even in trauma or revision surgeries with severe bone loss. 1 2 3 4 This is mainly true for juxta-articular primary bone tumors or metastasis affecting the proximal and distal femur, since these are two of the most common places for sarcomas or metastases to arise. 3 Furthermore, for patients presenting significant bone loss in the revision or trauma scenarios, modular megaprosthesis became a valuable way to manage these complex situations. 5 6 They provide the major advantage of modularity, which allows tailoring (to a certain extent) of the implant to the bone defect and patients' need. Additionally, long-term follow-up from these megaprosthesis already proved a reasonable implant survivorship with acceptable function. 7 8 9

Despite the notorious improvements regarding patients' preoperative clinical optimization, implants quality, surgical technique, and overall medical support in the perioperative period, surgical approach to resect bone tumors will always imply an aggressive nature, which coupled with patient's clinical complexity and extensive prosthetic reconstruction will increase wound-related complications, including surgical site infections (SSIs) and prosthetic joint infections (PJIs). 10 11 12

For primary oncologic implants, the general assumption is that the risk for prosthetic joint infection is around 5 to 10% based on studies with mid-term follow-up. 10 13 However, these numbers can be substantially higher, as highlighted in the PARITY randomized clinical trial, which reported a SSIs rate of 15 to 16.7%, for patients with bone tumors who underwent reconstruction with megaprosthesis. 14 To aggravate this scenario, infections occurring after bone tumor resection and reconstruction are particularly difficult to eradicate and carry a high risk of amputation, which has been reported to be around 36%. 13 15

Given all these facts, different strategies are employed in order to minimize infections in musculoskeletal oncology surgery, however, improvements are still needed. In this regard, our working group recently published results from the implementation of close incision negative-pressure wound therapy (ciNPWT) as a postoperative adjuvant method to minimize wound-related complications, in primary or metastatic bone tumors resections. 16 This study demonstrated lesser wound complications, in particular SSIs, for those patients where ciNPWT was used. However, one of the main criticism arrived from the heterogeneity within the cohort, which included several different diagnosis, and most importantly, different reconstructive techniques in a wide range of anatomic locations. 16 In order to better understand the preliminary findings elsewhere published, we promoted a study to assess the impact of ciNPWT as an adjuvant to minimize wound related complications, in patients with primary or metastatic bone tumors, exclusively located in the proximal or distal femur, and exclusively managed with wide resection and reconstruction with megaprosthesis.

## Materials and and Methods

### Patients

This observational retrospective study included a group of 41 patients diagnosed with localized primary bone sarcomas, bone giant cell tumors (GCT), or solitary metastatic bone disease exclusively located in the proximal or distal femur, with clinical indication for wide resection, between 2012 and 2021. A minimum 24-months postoperative follow-up was applied. All patients were identified from the Orthopedic Department database, and all underwent bone tumor wide surgical resection and reconstruction with modular megaprosthesis (proximal or distal femur) which were performed by one of two orthopedic oncology consultants (JSB or JP).

Patients were divided into two groups based on the postoperative surgical dressing applied: Group A (vacuum-assisted closure, VAC) received ciNPWT, and Group B (non-VAC) conventional dressings. The rational to use ciNPWT was based on the recent data showing that ciNPWT is helpful at reducing risk of wound complications after complex arthroplasty surgery. 17 18

No case within this cohort received preoperative radiotherapy in the surgical bed, however, patients with some bone sarcomas (in particular osteosarcoma) received pre- and postoperative chemotherapy. Patients with bone metastases received postoperative radiotherapy in the surgical bed, as well as standard medical therapy according to the subtype histology.

Data regarding postoperative wound-related complications, including wound dehiscence, persistent wound leakage, and SSIs, as well as causes for surgical revision within the first 2 postoperative years, were retrieved from patients' medical records. Postoperative complications were described according to Henderson et al. 19

In line with the standard protocol for musculoskeletal tumor surgery of our institution, surgical drains and perioperative intravenous antibiotics (cefazolin) were used in all patients, maintaining prophylactic antibiotherapy until drain removal, which means during 24 hours after surgery. Also, in order to study any other potential variables capable of promoting wound complications we included for further analysis pre- and postoperative hemoglobin levels and subsequent need for blood transfusion, preoperative total proteins count, preoperative glycemia, and surgical time for each case.

All data were subjected to anonymization in order to protect the privacy and confidentiality of participants. This study followed the ethical standards of the Declaration of Helsinki. Written and informed consent forms for surgical and clinical data collection for scientific purposes were obtained from all patients upon admission and before surgery, according to institutional protocol. A formal approval from our Institutional Review Board was also obtained for this study under number 132/24.

### Closed-Incision Negative-Pressure Wound Therapy (ciNPWT) Technique

**Fig. 1:**
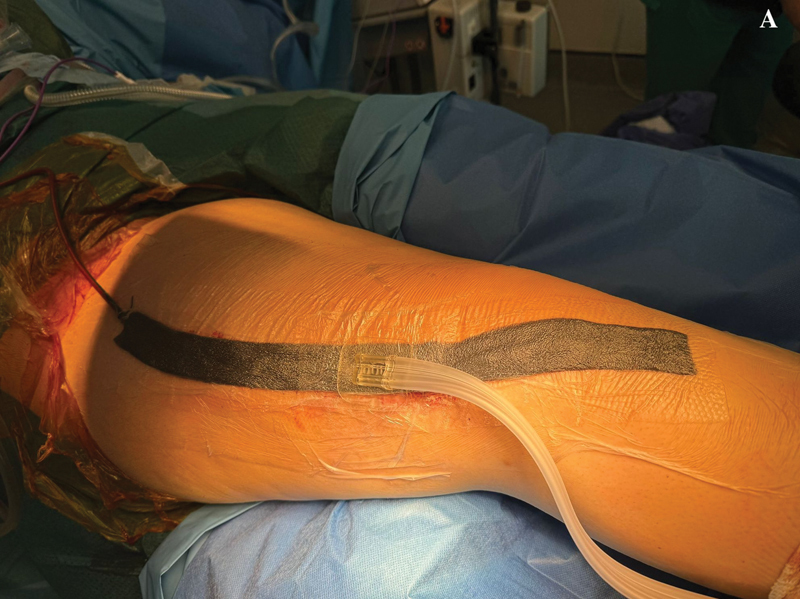
Immediate postoperative surgical wound with closed-incision negative-pressure wound therapy (ciNPWT) applied after a distal femur resection and reconstruction with megaprosthesis ( **A** ). We can also observe the drain in place together with the ciNPWT.

**Fig. 2:**
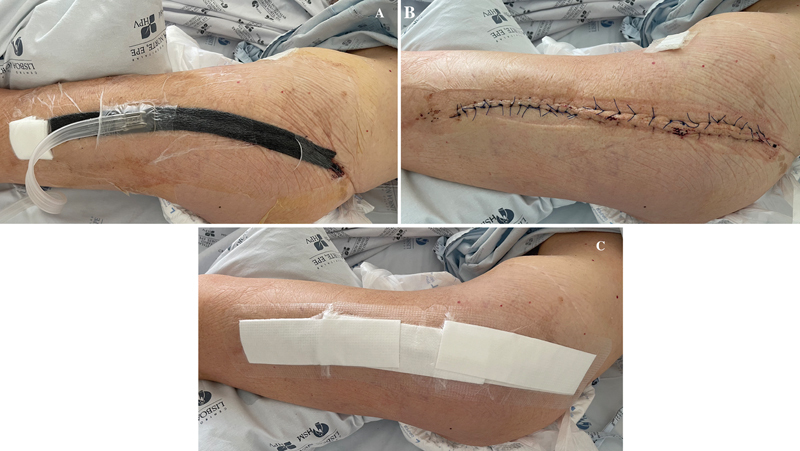
Postoperative surgical wound of a proximal femur resection and reconstruction with megaprosthesis with ciNPWT applied ( **A** ), after its removal at 1 week postsurgery ( **B** ), and with a conventional dressing applied ( **C** ).

After tumor resection, skeletal reconstruction with megaprosthesis and adequate soft tissue coverage, a drain is used to minimize risk for seroma and subsequent infection. Afterwords, the skin is closed with individual stitches separated by one centimeter using Ethilon (Ethicon, Inc., Bridgewater, NJ, USA) nylon 2.0 sutures. Vacuum is then applied to the drain and a Mepitel (Mölnlycke Health Care AB, Gothenburg, Sweden) stripe is applied over the closed surgical wound. Then, the negative pressure wound system is initiated, with a pressure value of 120 mmHg ( Figs. 1 , 2 ). The drain is usually removed 24 hours after surgery and negative pressure therapy is maintained during the first postoperative week, being subsequently replaced by a conventional dressing for another week ( Fig. 2 ).

### Statistical analysis

The statistical analysis was carried out using the commercial software package IBM SPSS Statistics for MAC OS X (IBM Corp., Armonk, NY, USA), version 24.0. All statistical tests were conducted for a significance level of 5%.

## Results

Patients' demographic and clinical features are depicted in Table 1 . For the cohort study of 41 patients, 20 (49%) were included in Group A, VAC; and 21 (51.0%) in Group B: non-VAC. The percentage of male gender was slightly higher for the VAC group, however, the small differences observed between genders were not significant ( *p* = 0.901). Also, despite the higher mean age in the VAC group compared with the non-VAC, these differences were not significant ( *p* = 0.275) ( Table 2 ).

**Table 1:** Patient demographics and clinical data

		VAC group (N = 20)		Non-VAC group (N = 21)		χ ^2^ or F	*p* -value
		N	%	N	%		
**Gender**	Male	8	40	8	38.1	χ ^2^ = 0.016	0.901
	Female	12	60	13	61.9		
**Diagnosis**	Bone sarcoma	6	30	8	38.1	F = 2.276	0.319
	Bone giant cell tumor	2	10	5	23.8		
	Bone metastasis	12	60	8	38.1		
**Location**	Proximal femur	13	65	10	47.6	χ ^2^ = 1.257	0.262
	Distal femur	7	35	11	52.4		
**Pathologic fracture**	Yes	11	55	4	19	χ ^2^ = 5.707	*0.017
	No	9	45	17	81		
**Fracture type**	Subcapital	1	5	2	9.5	F = 8.137	*0.028
	Supracondylar	3	15	1	4.8		
	Per and subtrochanteric	7	35	1	4.8		
**Reconstruction type**	Proximal femur megaprosthesis	13	65	10	47.6	χ ^2^ = 1.257	0.262
	Distal femur megaprosthesis	7	35	11	52.4		
**Cement**	Yes	12	60	10	47.6	χ ^2^ = 0.631	0.427
	No	8	40	11	52.4		
**Resection**	R0	12	60	13	61.9	F = 1.954	0.518
	R1	8	40	6	28.6		
	R2	0	0	2	9.5		

**Abbreviations:** χ ^2^ , Chi squared; VAC, vacuum-assisted closure. **Note:** * *p* ≤ 0.05.

**Table 2:** Relation between patients' age and the VAC and non-VAC groups

	VAC group (N = 20)		Non-VAC group (N = 21)		t _(39)_	*p* -value
	M	SD	M	SD		
**Age**	52.2	16.3	45.8	20.0	1.108	0.275

**Abbreviations:** M, mean; SD, standard deviation; VAC, vacuum-assisted closure.

Regarding diagnosis, the percentage of bone metastases was higher in the VAC group, with high percentages of bone sarcoma and giant cell tumor within the non-VAC group. Nonetheless, these differences were also not statistically significant ( *p* = 0.319). Again, the differences regarding tumor location or reconstruction with megaprosthesis were not significant ( *p* = 0.262). Similarly, the use of cemented or uncemented megaprosthesis revealed no significant differences ( *p* = 0.427). The percentage of pathologic fractures was higher in the VAC group, presenting statistically significant differences when compared with the non-VAC group ( *p* = 0.017).

Regarding the quality of bone tumor resection, the percentage of R0 was similar for both groups, while the percentage of R1 was higher for the VAC group and the percentage of R2 was higher for the non-VAC group. These differences were not statistically significant ( *p* = 0,518).

Overall, the majority of the cohorts presented no postoperative complications ( Table 3 ). Acute infection, classified as a complication type IVA by Henderson et al., 19 was observed in 4 cases (19%) within the non-VAC group, while only one case (5%) was recorded in the VAC group. Acute aseptic loosening (class IIA), chronic aseptic loosening (class IIB), mechanical failure of the implant (class IIIA) and local recurrence within the bone (class VA), all had one case each, with all these cases being within the non-VAC group. None of these complications presented significant differences between both groups ( *p* = 0.083).

**Table 3:** Contingency cross table for the relation between complicationsand the VAC and non-VAC groups

		VAC group (N = 20)		Non-VAC group (N = 21)		F	*p* -value
		N	%	N	%		
**Complications** 19	No	19	95.0	13	61.9	6.470	0.083
	Acute infection (IVA)	1	5.0	4	19.0		
	Acute aseptic loosening (IIA)	0	0	1	4.8		
	Chronic aseptic loosening (IIB)	0	0	1	4.8		
	Mechanical failure of the implant (IIIA)	0	0	1	4.8		
	Bone local recurrence (VA)	0	0	1	4.8		
**Wound dehiscence**	Yes	0	0	3	14.3	3.083	0.232
	No	20	100.0	18	85.7		
**Persistent fluid leakage**	Yes	0	0	3	14.3	3.083	0.232
	No	20	100.0	18	85.7		
**Surgical site infection**	Yes	1	5.0	4	19.0	1.888	0.343
	No	19	95.0	17	81.0		
**Need for surgical revision**	Yes	1	5.0	7	33.3	5.236	*0.045
	No	19	95.0	14	66.7		

**Abbreviation:** VAC, vacuum-assisted closure. **Note:** * *p* ≤ 0.05.

Wound dehiscence and persistent fluid leakage were higher for the non-VAC group, but without statistically significant differences. When analyzing specifically SSIs, which correspond to an acute infection, classified by Henderson et al. 19 as a type-IVA complication, this diagnosis occurred in 5% of the VAC and 19% of the non-VAC group. Although these differences were also not statistically significant ( *p* = 0.343), as shown in Table 4 .

**Table 4:** Contingency cross table for the relationship between diagnosis of surgical site infection and need for surgical revision

		VAC group (N = 20)		Non-VAC group (N = 21)		F	*p* -value
		N	%	N	%		
**Diagnosis of surgical site infection**	Yes	1	5.0	4	19.0	1.888	0.343
	No	19	95.0	17	81.0		
**Need for surgical revision**	Yes	1	5.0	7	33.3	5236	*0.045
	No	19	95.0	14	66.7		

**Abbreviation:** VAC, vacuum-assisted closure. **Note:** * *p* ≤ 0.05.

The variable “need for surgical revision” took into consideration all causes for revision within our cohort, meaning that were considered not only wound-related complications, but also any other conditions requiring a new surgical procedure. In this particular setting, the VAC group (5.0%) presented a lower need for surgical revision when compared with the non-VAC group (33.3%), with these differences being statistically significant ( *p* = 0.045), as shown in Table 4 .

The analysis of pre- and postoperative chemotherapy, postoperative radiotherapy, pre- and postoperative hemoglobin levels, the need for blood transfusion, the preoperative total proteins count, the preoperative glycemia, and the surgical time, also performed between the VAC and non-VAC groups. In none of those cases statistically significant differences were found ( Tables 5 6 ).

**Table 5:** Contingency cross table for the relation between pre- and postoperative chemotherapy, and postoperative radiotherapy

		VAC group (N = 20)		Non-VAC group (N = 21)		χ ^2^	*p* -value
		N	%	N	%		
**Preoperative Ct**	Yes	6	30.0	8	38.1	0.299	0.585
	No	14	70.0	13	61.9		
**Postoperative Ct**	Yes	10	50.0	11	52.4	0.023	0.879
	No	10	50.0	10	47.6		
**Postoperative RT**	Yes	10	50.0	7	33.3	1.172	0.279
	No	10	50.0	14	66.7		

**Abbreviations:** χ ^2^ , Chi squared; Ct, chemotherapy; RT, radiotherapy; VAC, vacuum-assisted closure.

**Table 6:** Contingency cross table for the relation between pre- and postoperative hemoglobin levels, the preoperative total proteins count, the preoperative and surgical time glycemia

	VAC group (N = 20)		Non-VAC group (N = 21)		t _(38)_	*p* -value
	M	SD	M	SD		
**Preoperative hemoglobin**	12.15	1.76	12.29	1.64	−0.251	0.803
**Postoperative hemoglobin**	8.64	1.37	9.27	0.99	−1.655	0.106
**Preoperative total protein count**	6.89	0.62	6.89	0.79	0.022	0.982
**Preoperative glycemia**	100.55	17.86	103.2	21.03	−0.429	0.670
**Surgical time (minutes)**	309.0	80.05	285.0	97.47	0.851	0.400

**Abbreviations:** M, mean; SD, standard deviation; VAC, vacuum-assisted closure.

A subsequent analysis of the cohort on the relation between all the variables failed to find any relation between the diagnosis of SSI and tumor subtype, anatomic location, presence of pathologic fracture, reconstruction type, the presence or not of cemented megaprosthesis, or quality of resection. However, the presence of wound dehiscence and persistent fluid leakage presented a clear positive correlation with the diagnosis of infection ( *p* ≤ 0.05), as shown in Table 7 .

**Table 7:** Contingency cross table for the relation between wound dehiscence and persistent fluid leakage and the diagnosis of surgical site infection (SSI)

		SSI diagnosis				F	*p* -value
		Yes (N = 5)		No (N = 36)			
		N	%	N	%		
**Wound dehiscence**	Yes	3	60	0	0	23.305	*0.001
	No	2	40	36	100		
**Persistent fluid** **leakage**	Yes	3	60	0	0	23.305	*0.001
	No	2	40	36	100		

**Note:** * *p* ≤ 0.05.

Again, the study of the relation between the variables and the need for surgical revision showed how the occurrence of complications, such as acute infection, chronic aseptic loosening, and local recurrence, together with the presence of wound dehiscence or persistent fluid leakage, indicate the need for a new surgical procedure ( Table 8 ).

**Table 8:** Contingency cross table for the relation between complications, wound dehiscence and persistent fluid leakage and the need for surgical revision

		Need for surgical revision				F	*p* -value
		Yes (N = 8)		No (N = 33)			
		N	%	N	%		
**Complications**	No	1	12.5	31	93.9	28.524	*0.000
	Acute infection	5	62.5	0	0		
	Acute aseptic loosening	0	0	1	3.0		
	Chronic aseptic loosening	1	12.5	0	0		
	Mechanical failure of the implant	0	0	1	3.0		
	Bone local recurrence	1	12.5	0	0		
**Wound dehiscence**	Yes	3	37.5	0	0	13.352	*0.005
	No	5	62.5	33	100		
**Persistent fluid leakage**	Yes	3	37.5	0	0	13.352	*0.005
	No	5	62.5	33	100		

**Note:** * *p* ≤ 0.05.

## Discussion

There is growing evidence for the advantages of using ciNPWT in hip and knee arthroplasties, in order to minimize wound-related complications. These advantages seem clearer for revision arthroplasties and for high-risk patients, when compared to conventional hip or knee prosthetic replacements. 20 21 22 23 Consequently, there is an increased use of ciNPWT in such clinical scenarios, whereas conventional dressings are still the standard procedure.

In bone tumor surgery, the evidence is less clear concerning the potential role of ciNPWT. A recent secondary analysis on the PARITY randomized clinical trial studied the impact of drain usage and negative pressure wound therapy as predictors for surgical site infection. 24 The conclusion highlighted that neither the use of postoperative drains nor the use of negative-pressure wound therapy was a predictor of surgical site infection. Batista et al. 25 published a recent study involving patients with malignant bone tumors, who underwent surgical resection and reconstruction with megaprosthesis using ciNPWT. These authors observed both lower rates of SSI and the absence of other complications such as dehiscence and fluid collections. 25

In this study we observed a higher number of overall complications, wound dehiscence, persistent fluid leakage and surgical site infection within the non-VAC group, however, the difference towards the VAC group was not statistically significant. As for the need for surgical revision, we observed statistically significant differences between the groups, however, these findings were only possible due to nonwound-related complications reported within the non-VAC group, which included patients treated before 2018, with a higher follow-up and necessarily increased opportunity for mechanical complications to develop. We also found a relation between the diagnosis of surgical site infection and presence of wound dehiscence or persistent fluid leakage within the overall cohort. The same was noted in the need for surgical revision, meaning this could be a consequence due to wound dehiscence, persistent fluid leakage, or SSIs.

Despite the lack of statistical superiority regarding minimizing wound-related complications in this study for cases using ciNPWT, we still found a favorable trend to improve results with this strategy: SSI of 5% within the VAC and 19% in the non-VAC group. This trend reinforces our belief in the advantages of this technique for patients with bone tumors, despite our cohort being limited in size, and needing a higher number of patients to enhance statistical power. Additionally, the results reported from the use of ciNPWT in orthopedic trauma and revisions arthroplasty points it as a valuable adjuvant for reducing risk of surgical site infection, dehiscence or seroma formation. 26 27

Musculoskeletal oncologic surgical procedures are particularly prone to infectious complications given the need for particularly wide dissections, complex bone and soft tissue reconstructions, significant blood loss, and prolonged surgical times. 28 To this day, the cornerstone for minimizing postoperative infections rate is perioperative antibiotic prophylaxis, but it seems insufficient given the high number of infections after tumor resections and reconstructive surgery. 15

Furthermore, there is still a lack of consensus regarding antibiotic prophylaxis after tumor resection and reconstruction with megaprosthesis, with the PARITY randomized clinical trial failing to prove differences between surgical site infection rate using different antibiotic regimens. 14 Despite this gap in knowledge, there are still few studies exploring options to improve postsurgical infection rates in musculoskeletal oncology. While ciNPWT is a promising option, it still has a very limited number of studies comparing the outcomes. Herein, we failed to prove with statistical significance the advantage of this technique in minimizing wound-related complications and, in particular, surgical site infections. However, we observed how the presence of wound dehiscence and persistent fluid leakage correlates with infection, and how these three parameters indicate the need to promote a new surgical procedure.

The authors acknowledge this study presents some limitations, such as its retrospective nature, low number of patients included, and some heterogeneity regarding the types of malignancy affecting the bone, which can all generate relevant bias in interpretation of the results. It is of utmost importance to promote multicentric prospective studies capable of generating more robust evidence in a larger population. Our working group is pursuing that goal and with time, we hope to better clarify the role of ciNPWT in malignant bone tumor surgery.

Wound-related complications and SSIs in particular have a potential catastrophic impact on patients with megaprosthesis after bone tumor resections. As such, measures capable of reducing these complication rates are urgently needed and all opportunities to improve results should be considered. The ciNPWT have showed a relevant potential in different settings including for musculoskeletal oncology surgery. 29 30 31 In our opinion, the findings herein presented still favor this strategy as an adjuvant to prevent wound-related complications and infection after proximal and distal femur bone tumor resection and reconstruction with megaprosthesis.

## Conclusion

Despite the reduction in wound dehiscence, persistent wound leakage, and surgical site infections when ciNPWT is used, there is a lack of statistical significance within the differences observed. Nonetheless, it seems clear that the presence of wound dehiscence and persistent wound leakage correlates well with surgical site infection; while wound dehiscence, persistent wound leakage, and surgical site infection correlate with the need for surgical revision.

There is a need for further prospective, multicentric, randomized studies to clarify the role of ciNPWT in the management of this cohort, as well as to measure the impact in preventing wound-related complications.
