# Supplementary material for: Closed-Incision Negative-Pressure Wound Therapy in Proximal and Distal Femur Megaprosthetic Reconstructions after Bone Tumor Resections
Source: Rev Bras Ortop (Sao Paulo). 2025 Apr 28;60(1):s00451802965. doi: 10.1055/s-0045-1802965 (PMC12037217; doi:10.1055/s-0045-1802965)
Supplement: Related texts

# Research article: Terapia de pressão negativa com a ferida encerrada em reconstruções do fémur proximal e distal com megapróteses após ressecções de tumores ósseos

**Keywords:** infecção da ferida cirúrgica, neoplasias, próteses, sarcoma, tratamento de ferimentos com pressão negativa

## Abstract

**Objetivo** O tratamento cirúrgico de tumores ósseos é agressivo por natureza e frequentemente seguido por complicações relacionadas com a ferida (CRFs). Para minimizar esses eventos, diferentes estratégias foram empregadas e o penso de pressão negativa ( *closed-incision negative-pressure wound therapy* , ciNPWT, em inglês) emergiu como possível adjuvante. Neste estudo, pretendemos avaliar o impacto dessa técnica na minimização de CRFs em pacientes com tumores de fêmur proximal e distal tratados com megapróteses.

**Métodos** Este estudo retrospectivo observacional incluiu 41 participantes diagnosticados com tumores de fêmur proximal ou distal tratados com ressecção alargada e reconstrução com megaprótese. Os pacientes foram divididos em dois grupos com base no penso cirúrgico pós-operatório aplicado: grupo de encerramento assistido por vácuo ( *vacuum-assisted closure* , VAC, em inglês), onde foi aplicado ciNPWT; e grupo não-VAC, que recebeu pensos convencionais. Dados sobre CRFs pós-operatórias e outras possíveis variáveis de interesse foram registados. A análise estatística foi realizada recorrendo ao programa IBM SPSS Statistics, versão 24.0.

**Resultados** Foram 20 pacientes incluídos no grupo VAC e 21 no grupo não-VAC. A maioria dos pacientes não apresentou complicações e não houve diferenças entre os grupos em termos de CRFs, incluindo infecção. No entanto, deiscência da ferida e extravasamento persistente de fluido tiveram correlação positiva com o diagnóstico de infecção que, juntos, apresentaram correlação com a necessidade de revisão cirúrgica.

**Conclusão** Apesar da ausência de significância estatística, a ciNPWT parece ajudar a minimizar a deiscência da ferida, o extravasamento persistente da ferida e as infecções da ferida operatória em pacientes com tumores ósseos do fêmur proximal e distal tratados com megaprótese. Além disso, a deiscência da ferida e o extravasamento persistente da ferida foram bem correlacionados com a infecção de ferida operatória, e os três parâmetros estão correlacionados com a necessidade de revisão cirúrgica.

## Introdução

A reconstrução óssea com megaprótese é uma abordagem comum após a ressecção de tumores ou mesmo cirurgias de trauma ou revisão com perda óssea extensa. 1 2 3 4 Isso se aplica particularmente a tumores ósseos primários justa-articulares ou metástases no fêmur proximal e distal, uma vez que estes são dois dos sítios mais comuns para o surgimento de sarcomas ou metástases. 3 Além disso, nos pacientes com perda óssea significativa em cenários de revisão ou trauma, as megapróteses modulares tornaram-se uma forma valiosa para tratamento dessas situações complexas. 5 6 Elas têm como principal vantagem a modularidade, que permite a adaptação (até certo ponto) do implante ao defeito ósseo e às necessidades dos pacientes. Ademais, o acompanhamento em longo prazo dessas megapróteses já provou uma sobrevida razoável do implante com função aceitável. 7 8 9

Apesar das notórias melhoras em relação à otimização clínica pré-operatória dos pacientes, à qualidade dos implantes, à técnica cirúrgica e ao suporte médico geral no período perioperatório, a abordagem cirúrgica para ressecção de tumores ósseos sempre implica em uma natureza agressiva, o que, somado à complexidade clínica do paciente e à extensa reconstrução protética, aumenta as complicações relacionadas à ferida, incluindo infecções do sítio cirúrgico (ISCs) e infecções da prótese articular (IPAs). 10 11 12

Nos implantes oncológicos primários, a suposição geral é que o risco de IPA é de cerca de 5 a 10% com base em estudos de acompanhamento em médio prazo. 10 13 No entanto, esses números podem ser substancialmente maiores, como destacado no estudo clínico randomizado PARITY, que relatou uma taxa de ISC de 15 a 16,7% em pacientes com tumores ósseos submetidos à reconstrução com megaprótese. 14 Agravando esse cenário, as infecções após a ressecção e reconstrução de tumores ósseos são particularmente difíceis de erradicar e apresentam alto risco de amputação, de cerca de 36%. 13 15

Diante desses fatos, diferentes estratégias são empregadas para minimizar infecções em cirurgias oncológicas musculoesqueléticas, mas melhoras ainda são necessárias. Nesse sentido, nosso grupo de trabalho publicou recentemente resultados da implementação da terapia de ferida de incisão fechada com pressão negativa ( *closed-incision negative-pressure wound therapy* , ciNPWT, em inglês) como método adjuvante pós-operatório para minimizar complicações em ressecções de tumores ósseos primários ou metastáticos. 16 Este estudo demonstrou menores taxas de complicações de feridas, em particular ISCs, nos pacientes submetidos à ciNPWT. No entanto, as principais críticas foram a heterogeneidade dentro da coorte, que incluiu diversos diagnósticos e, mais importante, as diferentes técnicas reconstrutivas em uma ampla gama de localizações anatômicas. 16 Para entender melhor os resultados preliminares publicados em outros lugares, realizamos um estudo para avaliação do impacto da ciNPWT como adjuvante para minimizar complicações relacionadas à ferida em pacientes com tumores ósseos primários ou metastáticos localizados exclusivamente no fêmur proximal ou distal e tratados apenas com ressecção ampla e reconstrução com megaprótese.

## Materiais e Métodos

## Pacientes

Este estudo retrospectivo observacional incluiu um grupo de 41 pacientes diagnosticados com sarcomas ósseos primários localizados, tumores ósseos de células gigantes (TCG) ou doença óssea metastática solitária exclusivamente no fêmur proximal ou distal com indicação clínica para ressecção ampla entre 2012 e 2021. O acompanhamento pós-operatório mínimo foi de 24 meses. Todos os pacientes foram identificados no banco de dados do Departamento de Ortopedia e submetidos à ressecção cirúrgica ampla do tumor ósseo e à reconstrução com megaprótese modular (fêmur proximal ou distal). Estes procedimentos foram executados por um dos dois consultores de oncologia ortopédica (JSB ou JP).

Os pacientes foram divididos em dois grupos com base no curativo cirúrgico pós-operatório aplicado: Grupo A (fechamento assistido por vácuo [ *vacuum-assisted closure* , VAC, em inglês]), submetido à ciNPWT, e Grupo B (não-VAC), que recebeu curativos convencionais. A justificativa para uso de ciNPWT foi baseada em dados recentes que mostram que esta modalidade auxilia a redução do risco de complicações da ferida após artroplastias complexas. 17 18

Nenhum caso desta coorte recebeu radioterapia pré-operatória no leito cirúrgico. No entanto, pacientes com alguns sarcomas ósseos (em particular osteossarcoma) receberam quimioterapia pré- e pós-operatória. Pacientes com metástases ósseas receberam radioterapia pós-operatória no leito cirúrgico, juntamente com terapia médica padrão de acordo com a histologia do subtipo tumoral.

Dados sobre complicações pós-operatórias relacionadas à ferida, incluindo deiscência da ferida, extravasamento persistente da ferida e ISCs, bem como causas de revisão cirúrgica nos 2 primeiros anos pós-operatórios, foram recuperados dos prontuários médicos dos pacientes. As complicações pós-operatórias foram descritas de acordo com Henderson et al. 19

Seguindo o protocolo padrão de cirurgias de tumores musculoesqueléticos de nossa instituição, todos os pacientes receberam drenos cirúrgicos e antibióticos intravenosos perioperatórios (cefazolina); a antibioterapia profilática foi mantida até a remoção do dreno, ou seja, 24 horas após a cirurgia. Além disso, para estudar quaisquer outras possíveis variáveis capazes de promover complicações da ferida, incluímos para análise posterior os níveis de hemoglobina pré e pós-operatórios e subsequente necessidade de transfusão de sangue, as concentrações de proteínas totais pré-operatórias, a glicemia pré-operatória e o tempo cirúrgico de cada caso.

Todos os dados foram submetidos à anonimização para proteger a privacidade e a confidencialidade dos participantes. Este estudo seguiu os padrões éticos da Declaração de Helsinque. Termos de consentimento livre e esclarecido para coleta de dados cirúrgicos e clínicos para fins científicos foram assinados por todos os pacientes à internação e antes da cirurgia, de acordo com o protocolo institucional. Este estudo recebeu a aprovação formal do nosso Conselho de Revisão Institucional sob o número 132/24.

## Técnica de terapia de ferida de incisão fechada com pressão negativa (ciNPWT)

Após a ressecção do tumor, a reconstrução esquelética com megaprótese e a cobertura adequada de tecido mole, um dreno foi usado para minimizar o risco de seroma e infecção subsequente. Depois, a pele foi fechada com pontos simples separados por um centímetro com suturas de Nylon 2.0 Ethilon (Ethicon, Inc., Bridgewater, NJ, EUA). O vácuo foi então aplicado ao dreno e uma tira de Mepitel (Mölnlycke Health Care AB, Gotemburgo, Suécia) foi colocado sobre a ferida cirúrgica fechada. A seguir, o sistema de ferida de pressão negativa foi iniciado, com pressão de 120 mmHg ( Figs. 1 , 2 ). O dreno foi geralmente retirado 24 horas após a cirurgia e a terapia por pressão negativa foi mantida durante a primeira semana pós-operatória, sendo posteriormente substituída por um curativo convencional por mais 1 semana ( Fig. 2 ).

**Fig. 1:** Ferida cirúrgica no período pós-operatório imediato com terapia de ferida de incisão fechada com pressão negativa ( *closed-incision negative-pressure wound therapy* , ciNPWT, em inglês) aplicada após ressecção do fêmur distal e reconstrução com megaprótese ( **A** ). Note o dreno colocado junto com a ciNPWT.

**Fig. 2:** Ferida cirúrgica pós-operatória de ressecção e reconstrução do fêmur proximal com megaprótese com terapia de ferida de incisão fechada com pressão negativa (ciNPWT) aplicada ( **A** ), após sua remoção com 1 semana de pós-cirurgia ( **B** ) e com curativo convencional aplicado ( **C** ).

## Análise estatística

A análise estatística foi realizada usando o software comercial IBM SPSS Statistics for MAC OS X (IBM Corp., Armonk, NY, EUA), versão 24.0. Todos os testes estatísticos foram conduzidos com nível de significância de 5%.

## Resultados

As características demográficas e clínicas dos pacientes são descritas na Tabela 1 . Da coorte do estudo de 41 pacientes, 20 (49%) foram incluídos no Grupo A, VAC; e 21 (51,0%) no Grupo B: não-VAC. O número de homens foi ligeiramente maior no grupo VAC, mas pequenas diferenças observadas entre os gêneros não foram significativas ( *p* = 0,901). Além disso, apesar da maior média de idade no grupo VAC em comparação ao não-VAC, essas diferenças não foram significativas ( *p* = 0,275) ( Tabela 2 ).

**Tabela 1:** Dados demográficos e clínicos dos pacientes

		Grupo VAC (N = 20)		Grupo não-VAC (N = 21)		χ ^2^ ou F	Valor de *p*
		N	%	N	%		
**Sexo**	Masculino	8	40,0	8	38,1	χ ^2^ = 0,016	0,901
	Feminino	12	60,0	13	61,9		
**Diagnóstico**	Sarcoma ósseo	6	30,0	8	38,1	F = 2,276	0,319
	Tumor de células gigantes ósseas	2	10,0	5	23,8		
	Metástase óssea	12	60,0	8	38,1		
**Localização**	Fêmur proximal	13	65,0	10	47,6	χ ^2^ = 1,257	0,262
	Fêmur distal	7	35,0	11	52,4		
**Fratura patológica**	Sim	11	55,0	4	19,0	χ ^2^ = 5,707	*0,017
	Não	9	45,0	17	81,0		
**Tipo de fratura**	Subcapital	1	5,0	2	9,5	F = 8,137	*0,028
	Supracondilar	3	15,0	1	4,8		
	Per e subtrocantérica	7	35,0	1	4,8		
**Tipo de reconstrução**	Megaprótese do fêmur proximal	13	65,0	10	47,6	χ ^2^ = 1,257	0,262
	Megaprótese do fêmur distal	7	35,0	11	52,4		
**Cimento**	Sim	12	60,0	10	47,6	χ ^2^ = 0,631	0,427
	Não	8	40,0	11	52,4		
**Ressecção (R)**	R0	12	60,0	13	61,9	F = 1,954	0,518
	R1	8	40,0	6	28,6		
	R2	0	0,0	2	9,5		

**Abreviaturas:** χ ^2^ , Qui ao quadrado; VAC, *vacuum-assisted closure* (fechamento assistido por vácuo). **Nota:** * *p* ≤ 0,05.

**Tabela 2:** Relação entre a idade do paciente e os grupos VAC e não-VAC

	Grupo VAC (N = 20)		Grupo não-VAC (N = 21)		t _(39)_	Valor de *p*
	M	DP	M	DP		
**Idade**	52,2	16,3	45,8	20,0	1,108	0,275

Abreviaturas: DP, desvio padrão; M, média; VAC, *vacuum-assisted closure* (fechamento assistido por vácuo).

Em relação ao diagnóstico, a porcentagem de metástases ósseas foi maior no grupo VAC, com altas porcentagens de sarcoma ósseo e tumor de células gigantes no grupo não-VAC. Porém, essas diferenças também não foram estatisticamente significativas ( *p* = 0,319). Novamente, as diferenças quanto à localização do tumor ou reconstrução com megaprótese não foram significativas ( *p* = 0,262). Da mesma forma, o uso de megaprótese cimentada ou não cimentada não revelou diferenças significativas ( *p* = 0,427). A porcentagem de fraturas patológicas foi maior no grupo VAC, sendo estatisticamente significativa em comparação ao grupo não-VAC ( *p* = 0,017).

Em relação à qualidade da ressecção do tumor ósseo, a porcentagem de ressecção (R) 0 foi semelhante em ambos os grupos, enquanto a porcentagem de R1 foi maior no grupo VAC e a porcentagem de R2 foi maior no grupo não VAC. Essas diferenças não foram estatisticamente significativas ( *p* = 0,518).

A maioria da coorte não apresentou complicações pós-operatórias ( Tabela 3 ). Infecções agudas, classificadas como complicação de tipo IVA segundo Henderson et al., 19 foram observadas em 4 casos (19%) do grupo não-VAC e em apenas um caso (5%) do VAC. O afrouxamento asséptico agudo (classe IIA), o afrouxamento asséptico crônico (classe IIB), a falha mecânica do implante (classe IIIA) e a recidiva óssea local (classe VA), todos tiveram um caso cada, todos do grupo não VAC. Nenhuma dessas complicações apresentou diferenças significativas entre os dois grupos ( *p* = 0,083).

**Tabela 3:** Tabela de contingência cruzada para a relação entre complicações e os grupos VAC e não-VAC

		Grupo VAC (N = 20)		Grupo não-VAC (N = 21)		F	Valor de *p*
		N	%	N	%		
** Complicações 19**	Não	19	95	13	61,9	6,470	0,083
	Infecção aguda (IVA)	1	5	4	19,0		
	Afrouxamento asséptico agudo (IIA)	0	0	1	4,8		
	Afrouxamento asséptico crônico (IIB)	0	0	1	4,8		
	Falência mecânica do implante (IIIA)	0	0	1	4,8		
	Recidiva óssea local (VA)	0	0	1	4,8		
**Deiscência da ferida**	Sim	0	0	3	14,3	3,083	0,232
	Não	20	100	18	85,7		
**Extravasamento persistente de fluido**	Sim	0	0	3	14,3	3,083	0,232
	Não	20	100	18	85,7		
**Infecção do sítio cirúrgico**	Sim	1	5	4	19,0	1,888	0,343
	Não	19	95	17	81,0		
**Necessidade de revisão cirúrgica**	Sim	1	5	7	33,3	5,236	*0,045
	Não	19	95	14	66,7		

**Abreviatura:** VAC, *vacuum-assisted closure* (fechamento assistido por vácuo). **Nota:** * *p* ≤ 0,05.

A deiscência de ferida e o extravasamento persistente de fluido foram mais frequentes no grupo não-VAC, mas sem diferenças estatisticamente significativas. As ISCs, que correspondem a uma infecção aguda classificada por Henderson et al. 19 como complicação do tipo IVA, ocorreram em 5% do grupo VAC e 19% do não-VAC, mas essas diferenças não foram estatisticamente significativas ( *p* = 0,343), como mostrado na Tabela 4 .

**Tabela 4:** Tabela de contingência cruzada para a relação diagnóstico de infecção do sítio cirúrgico e necessidade de revisão cirúrgica

		Grupo VAC (N = 20)		Grupo não-VAC (N = 21)		F	Valor de *p*
		N	%	N	%		
**Diagnóstico de infecção do sítio cirúrgico**	Sim	1	5,0	4	19,0	1,888	0,343
	Não	19	95,0	17	81,0		
**Necessidade de revisão cirúrgica**	Sim	1	5,0	7	33,3	5,236	*0,045
	Não	19	95,0	14	66,7		

**Abreviatura:** VAC, *vacuum-assisted closure* (fechamento assistido por vácuo). **Nota:** * *p* ≤ 0,05.

A variável “necessidade de revisão cirúrgica” considerou todas as causas de revisão dentro da nossa coorte, ou seja, não apenas complicações relacionadas à ferida, mas também quaisquer outras condições que exigissem um novo procedimento cirúrgico. Neste cenário específico, o grupo VAC (5,0%) apresentou menor necessidade de revisão cirúrgica em comparação ao não-VAC (33,3%), sendo essa diferença estatisticamente significativa ( *p* = 0,045), como pode ser visto na Tabela 4 .

As análises da quimioterapia pré- e pós-operatória, radioterapia pós-operatória, níveis de hemoglobina pré- e pós-operatória, necessidade de transfusão sanguínea, concentração de proteínas totais pré-operatórias, glicemia pré-operatória e tempo cirúrgico também foram realizada entre os grupos VAC e não-VAC. Em nenhum desses casos houve diferença estatisticamente significativa ( Tabelas 5 6 ).

**Tabela 5:** Tabela de contingência cruzada da relação entre quimioterapia e radioterapia pré e pós-operatórias

		Grupo VAC (N = 20)		Grupo não-VAC (N = 21)		χ ^2^	Valores de *p*
		N	%	N	%		
**QT pré-operatória**	Sim	6	30,0	8	38,1	0,299	0,585
	Não	14	70,0	13	61,9		
**QT pós-operatória**	Sim	10	50,0	11	52,4	0,023	0,879
	Não	10	50,0	10	47,6		
**RT pós-operatória**	Sim	10	50,0	7	33,3	1,172	0,279
	Não	10	50,0	14	66,7		

Abreviaturas: χ ^2^ , Qui ao quadrado; QT, quimioterapia; RT, radioterapia; VAC, *vacuum-assisted closure* (fechamento assistido por vácuo).

**Tabela 6:** Tabela de contingência cruzada da relação entre os níveis pré e pós-operatórios de hemoglobina, as concentrações pré-operatórias de proteínas totais, a glicemia pré-operatória

	Grupo VAC (N = 20)		Grupo não-VAC (N = 21)		t _(38)_	Valores de *p*
	M	DP	M	DP		
**Hemoglobina pré-operatória**	12,15	1,76	12,29	1,64	−0,251	0,803
**Hemoglobina pós-operatória**	8,64	1,37	9,27	0,99	−1,655	0,106
**Concentração pré-operatória de proteínas totais**	6,89	0,62	6,89	0,79	0,022	0,982
**Glicemia pré-operatória**	100,55	17,86	103,20	21,03	−0,429	0,670
**Tempo cirúrgico (minutos)**	309,00	80,05	285,00	97,47	0,851	0,400

Abreviaturas: DP, desvio padrão; M, média; VAC, *vacuum-assisted closure* (fechamento assistido por vácuo).

Uma análise subsequente da coorte para estudo da relação entre todas as variáveis avaliadas não encontrou qualquer associação entre o diagnóstico de ISC e subtipo de tumor, a localização anatômica, a presença de fratura patológica, o tipo de reconstrução, a presença ou não de megaprótese cimentada, ou a qualidade da ressecção. No entanto, a presença de deiscência da ferida e extravasamento persistente de fluido apresentou uma clara correlação positiva com o diagnóstico de infecção ( *p* ≤ 0,05), como mostrado na Tabela 7 .

**Tabela 7:** Tabela de contingência cruzada da relação entre deiscência da ferida e extravasamento persistente de fluido e o diagnóstico de infecção do sítio cirúrgico (ISC)

		Diagnóstico de ISC				F	Valor de *p*
		Sim (N = 5)		Não (N = 36)			
		N	%	N	%		
**Deiscência da ferida**	Sim	3	60	0	0	23,305	*0,001
	Não	2	40	36	100		
**Extravasamento persistente de fluido**	Sim	3	60	0	0	23,305	*0,001
	Não	2	40	36	100		

**Nota:** * *p* ≤ 0,05.

Mais uma vez, o estudo da relação entre as variáveis e a necessidade de revisão cirúrgica mostrou como a ocorrência de complicações, como infecção aguda, afrouxamento asséptico crônico e recidiva local, juntamente com a presença de deiscência de ferida ou extravasamento persistente de fluido, são indicativos da necessidade de novo procedimento cirúrgico. ( Tabela 8 ).

**Tabela 8:** Tabela de contingência cruzada da relação entre complicações, deiscência da ferida e extravasamento persistente de fluido e a necessidade de revisão cirúrgica

		Necessidade de revisão cirúrgica				F	Valor de *p*
		Sim (N = 8)		Não (N = 33)			
		N	%	N	%		
**Complicações**	Não	1	12,5	31	93,9	28,524	*0,000
	Infecção aguda	5	62,5	0	0,0		
	Afrouxamento asséptico agudo	0	0,0	1	3,0		
	Afrouxamento asséptico crônico	1	12,5	0	0,0		
	Falência mecânica do implante	0	0,0	1	3,0		
	Recidiva óssea local	1	12,5	0	0,0		
**Deiscência da ferida**	Sim	3	37,5	0	0,0	13,352	*0,005
	Não	5	62,5	33	100,0		
**Extravasamento persistente de fluido**	Sim	3	37,5	0	0,0	13,352	*0,005
	Não	5	62,5	33	100,0		

**Nota:** * *p* ≤ 0,05.

## Discussão

Há evidências crescentes das vantagens do uso de ciNPWT em artroplastias de quadril e joelho para minimizar as complicações relacionadas à ferida. Essas vantagens parecem mais claras nas artroplastias de revisão e em pacientes de alto risco em comparação às artroplastias de quadril ou joelho. 20 21 22 23 Consequentemente, há um aumento no uso de ciNPWT em tais cenários clínicos, embora curativos convencionais ainda sejam o procedimento padrão.

Na cirurgia de tumor ósseo, as evidências quanto ao possível papel da ciNPWT são menos claras. Uma análise secundária recente do estudo clínico randomizado PARITY avaliou o impacto do uso de dreno e da terapia de pressão negativa na ferida como fatores preditivos de ISC. 24 A conclusão destacou que nem o uso de drenos pós-operatórios nem o uso de terapia de pressão negativa na ferida foram fatores preditivos de ISC. Batista et al. 25 publicaram um estudo recente sobre pacientes com tumores ósseos malignos submetidos à ressecção cirúrgica, reconstrução com megaprótese e uso de ciNPWT. Esses autores observaram tanto menores taxas de ISC quanto ausência de outras complicações, como deiscências e coleções fluidas. 25

Neste estudo, observamos um número maior de complicações gerais, deiscência da ferida, extravasamento persistente de fluido e ISC no grupo não-VAC; no entanto, a diferença em relação ao grupo VAC não foi estatisticamente significativa. Quanto à necessidade de revisão cirúrgica, observamos diferenças estatisticamente significativas entre os grupos, mas esses achados só foram possíveis devido a complicações não relacionadas à ferida relatadas no grupo não-VAC, que incluiu pacientes tratados antes de 2018, com um acompanhamento maior e necessariamente maior oportunidade de desenvolvimento de complicações mecânicas. Também observamos uma relação entre o diagnóstico de ISC e a presença de deiscência da ferida ou extravasamento persistente de fluido dentro da coorte geral. O mesmo foi notado na necessidade de revisão cirúrgica, que pode ser consequência de deiscência da ferida, extravasamento persistente de fluido ou ISCs.

Apesar da ausência de superioridade estatística em relação à minimização de complicações relacionadas à ferida neste estudo nos casos com ciNPWT, ainda encontramos uma tendência favorável a melhores resultados com esta estratégia: ISC de 5% no grupo VAC e 19% no não-VAC. Esta tendência reforça nossa crença nas vantagens dessa técnica para pacientes com tumores ósseos, apesar de nossa coorte ter tamanho limitado e haver necessidade de um número maior de pacientes para aumento do poder estatístico. Além disso, os resultados relatados do uso de ciNPWT em trauma ortopédico e artroplastia de revisão apontam que esta técnica é um adjuvante valioso para reduzir o risco de ISC, deiscência ou formação de seroma. 26 27

Os procedimentos cirúrgicos oncológicos musculoesqueléticos são particularmente propensos a complicações infecciosas devido à necessidade de dissecções bastante amplas, reconstruções complexas de ossos e tecidos moles, perda significativa de sangue e tempos cirúrgicos prolongados. 28 Até hoje, a pedra angular para minimizar a taxa de infecções pós-operatórias é a profilaxia antibiótica perioperatória, apesar de parecer insuficiente dado o alto número de infecções após ressecções tumorais e cirurgias reconstrutivas. 15

Ademais, ainda não há consenso quanto à profilaxia antibiótica após a ressecção e a reconstrução tumoral com megapróteses; o estudo clínico randomizado PARITY não conseguiu comprovar diferenças entre a taxa de ISC com diferentes regimes antibióticos. 14 Apesar dessa lacuna no conhecimento, ainda há poucos estudos explorando opções para melhorar as taxas de infecção pós-cirúrgica em oncologia musculoesquelética. Enquanto a ciNPWT é uma opção promissora, o número de estudos com comparação de resultados ainda é muito limitado. Aqui, falhamos em provar com significância estatística a vantagem dessa técnica em minimizar complicações relacionadas à ferida e, em particular, ISCs. No entanto, observamos como a presença de deiscência da ferida e extravasamento persistente de fluido se correlacionam com a infecção e como esses três parâmetros indicam a necessidade de realização de um novo procedimento cirúrgico.

Os autores reconhecem que este estudo apresenta algumas limitações, como sua natureza retrospectiva, o baixo número de pacientes incluídos e certa heterogeneidade quanto aos tipos de malignidade que acometem o osso, podendo gerar um viés relevante na interpretação dos resultados. É de suma importância promover estudos prospectivos multicêntricos capazes de gerar evidências mais robustas em uma população maior. Nosso grupo de trabalho está buscando esse objetivo e, com o tempo, esperamos esclarecer melhor o papel da ciNPWT na cirurgia de tumor ósseo maligno.

Complicações relacionadas a feridas e ISCs em particular têm um possível impacto catastrófico em pacientes com megapróteses após ressecções de tumores ósseos. Como tal, medidas que possam reduzir essas taxas de complicações são urgentemente necessárias e todas as oportunidades para melhorar os resultados devem ser consideradas. A ciNPWT mostrou um potencial relevante em diferentes cenários, inclusive na cirurgia oncológica musculoesquelética. 29 30 31 Em nossa opinião, os achados aqui apresentados ainda favorecem esta estratégia como adjuvante na prevenção de complicações e infecções relacionadas à ferida após a ressecção e reconstrução de tumores ósseos do fêmur proximal e distal com megapróteses.

## Conclusão

Apesar da redução na deiscência da ferida, extravasamento persistente da ferida e ISCs com o uso de ciNPWT, há uma falta de significância estatística nas diferenças observadas. No entanto, parece claro que a presença de deiscência da ferida e extravasamento persistente da ferida é bem correlacionada a ISCs, enquanto a deiscência da ferida, o extravasamento persistente da ferida e a ISC são bem correlacionadas à necessidade de revisão cirúrgica.

Estudos prospectivos, multicêntricos e randomizados são necessários para esclarecer o papel da ciNPWT no tratamento desses pacientes, assim como para medir seu impacto na prevenção de complicações relacionadas à ferida.
